# Neuromelanin, iron and MRI measurements in midbrain tissues of Parkinson’s and Alzheimer’s subjects

**DOI:** 10.3389/fnagi.2026.1672578

**Published:** 2026-02-04

**Authors:** Fabio A. Zucca, Clifford M. Cassidy, Michela Sturini, Lauri Tuominen, Fulvio Adorni, Victoria Cheung, Luigi Casella, David Sulzer, Gianni Pezzoli, Ioannis U. Isaias, Guillermo Horga, Luigi Zecca

**Affiliations:** 1 Institute of Biomedical Technologies, National Research Council of Italy, Segrate, Italy; 2 Institute of Mental Health Research at the Royal, University of Ottawa, Ottawa, ON, Canada; 3 Renaissance School of Medicine at Stony Brook University, Stony Brook, NY, United States; 4 Department of Chemistry, University of Pavia, Pavia, Italy; 5 Departments of Psychiatry, Neurology, and Pharmacology, Columbia University Medical Center, New York, NY, United States; 6 Division of Molecular Therapeutics, New York State Psychiatric Institute, New York, NY, United States; 7 Parkinson Institute of Milan, ASST G. Pini-CTO, Milan, Italy; 8 Pezzoli Foundation for Parkinson’s Disease, Milan, Italy; 9 Department of Neurology, University Hospital and Julius Maximilian University, Würzburg, Germany; 10 Department of Psychiatry, Columbia University, New York, NY, United States; 11 Division of Translational Imaging, New York State Psychiatric Institute, New York, NY, United States

**Keywords:** Alzheimer’s disease, imaging map, iron, magnetic resonance imaging, neuromelanin, Parkinson’s disease

## Abstract

**Background:**

In Parkinson’s disease, decreased neuromelanin and increased Fe concentrations derive from the loss of neuromelanin-containing neurons in the substantia nigra, which is spared in Alzheimer’s disease. We aimed to measure neuromelanin and Fe concentrations in post mortem midbrain subregions of patients with Parkinson’s and Alzheimer’s disease, and to investigate their effect on MRI signals.

**Methods:**

We imaged neuromelanin in brain slices of Parkinson’s (*N* = 4) and Alzheimer’s disease (*N* = 7) subjects using neuromelanin-sensitive MRI sequences, compared the results with neuromelanin and Fe concentrations in the same sections, and calculated imaging maps for each subject.

**Results:**

Both neuromelanin and Fe concentrations provided unique contributions to neuromelanin-MRI. Compared to Alzheimer’s disease, subjects with Parkinson’s disease exhibited increased neuromelanin-MRI values in the superior colliculus and periaqueductal gray matter, and higher Fe concentration in the substantia nigra.

**Conclusion:**

Results support the use of neuromelanin-MRI to study neuromelanin and Fe in substantia nigra and neighboring regions for investigating patients with Parkinson’s disease and other movement disorders.

## 1 Introduction

In the healthy human brain, neuromelanin (NM) is always associated with large amounts of Fe and other metals (Zecca et al., 2004, 2008a). NM is a dark brown-black, insoluble polymer that accumulates during aging in specific intraneuronal organelles (Zucca et al., 2018), together with lipid bodies, proteins and metals (Zecca et al., 2008a). In particular, NM is most abundant in dopaminergic neurons of the substantia nigra (SN) and in noradrenergic neurons of the locus coeruleus (Zecca et al., 2004; Wakamatsu et al., 2015; Sulzer et al., 2018; Monzani et al., 2019), as well as in other brain areas, albeit in smaller amounts (Zecca et al., 2008a; Engelen et al., 2012). In Parkinson’s disease (PD), there is a loss of NM-containing dopaminergic neurons of the SN (Kastner et al., 1992), leading to a marked decrease of NM concentration (Zecca et al., 2002), and an increase in both total Fe and NM-bound Fe content (Dexter et al., 1989; Riederer et al., 1989; Jellinger et al., 1992; Brooks et al., 2020). NM is consistently associated with Fe in variable amounts depending on the brain region, while Fe can obviously exist in combination with many types of molecules as high- or low-stability complexes (Zecca et al., 2004; Zecca et al., 2008a; Ward et al., 2014). In Alzheimer’s disease (AD), the SN neurons are not so heavily affected as in PD (Zarow et al., 2003), showing unchanged levels of NM concentration (Cai et al., 2023), but higher neuronal loss and Fe deposition in cortical regions (Ward et al., 2014; Möller et al., 2019).

An accurate method to quantitatively image these features of PD would help to diagnose the disease, evaluate its progression, and assess responses to therapy. MRI-based methods have become attractive tools to measure NM and Fe content (Reimão et al., 2016; Sulzer et al., 2018; Möller et al., 2019; Jin et al., 2022; Liu et al., 2024). MRI sequences sensitive to regional variability in NM concentrations (NM-MRI) can quantitatively detect NM in the SN (Cassidy et al., 2019), and reveal the decrease of NM content, which is a direct consequence of the loss of dopaminergic pigmented neurons in PD subjects (Isaias et al., 2016; Sulzer et al., 2018; Gaurav et al., 2021). Quantitative susceptibility mapping is an MRI method for imaging Fe deposition and can demonstrate Fe accumulation in the SN of PD patients, which is related to disease progression (Gaurav et al., 2025).

However, the contrast mechanism is not yet fully clear, and questions remain, including the independent influences of NM and Fe on NM-MRI contrast (Trujillo et al., 2017). To address this question, we have measured NM and Fe concentrations in the midbrain of subjects with AD and PD, and evaluated the relationship between their concentration and the NM-MRI signal.

## 2 Methods

### 2.1 Study design

The post mortem specimens of human midbrain tissue were obtained from the New York Brain Bank at Columbia University. Eleven specimens were obtained from donors (from 44 until >88 years at the time of death), specifically from individuals who suffered from AD or other forms of dementia (*N* = 7) and from individuals who suffered from PD with dementia (*N* = 4) at the time of death (Supplementary Table S1). The younger male subject (44 years of age) was retained, despite his substantial age difference, as his data were within the same range as those of the other subjects, and exclusion of this subject had little impact on the results.

### 2.2 Brain tissue preparation and imaging scanning conditions

Specimens were ~3 mm-thick sections dissected between the rostral and caudal parts from frozen tissue of right hemi-midbrain of each subject: demographic and clinical information of subjects with AD or other dementia and PD are reported in Supplementary Table S1. These slices contained the SN and were stored at −80 °C until use.

For the MRI scanning sessions, the specimens were progressively thawed to 20 °C, as verified with a laser thermometer, and scanned using NM-MRI sequences. Specimens were placed in a custom-made dish 3D-printed from MRI-compatible nylon polymer (NW Rapid Mfg, McMinnville, OR, USA), and a matching grid-insert lid was placed on top and affixed to hold the specimen in place (Supplementary Figure S1). While secured in the dish, specimens were fully immersed in an MRI-invisible lubricant (Fomblin^®^ perfluoropolyether Y25; Solvay, Thorofare, NJ, USA) and placed in a desiccator for 30 min to remove air from the tissue. Wells at the four cardinal points of the rim of the dish were filled with water to mark its location and orientation in the MRI images. For further description of methods, see our prior report (Cassidy et al., 2019).

MR images of the specimens were acquired using a GE Healthcare 3 T MR750 scanner. The dishes containing the specimens were placed on a custom stand inside a 32-channel, phased-array Nova head coil and scanned using the 2D GRE-MT NM-MRI sequence. Parameters were as follows: repetition time (TR) = 260 ms; echo time (TE) = 2.68 ms; flip angle = 40°; in-plane resolution = 0.3125 × 0.3125 mm^2^; field of view (FoV) = 160 × 80; slice thickness = 0.60 mm; slice gap = 0 mm; magnetization transfer frequency offset = 1,200 Hz; number of excitations (NEX) = 8. The processing pipeline for NM-MRI data included averaging across slices to flatten the images into 2D images. This flattening removed the third (thickness) dimension, thereby allowing comparison with concentration estimates from the dissected tissue, which included the full slice thickness.

After the scanning session, samples were refrozen in place and marked with gridlines by applying methylene blue dye (0.05% water solution; Sigma-Aldrich, St. Louis, MO, USA) to the tissue using the grid insert as a stamp (Supplementary Figure S1). Guides built into the dish walls ensured that the orientation of the grid relative to the specimen was fixed at all times. For each specimen, grid sections were assigned to specific midbrain regions based on visual inspection of NM-MRI images according to accurate anatomical criteria. We focused our attention on the SN and neighboring regions: crus cerebri (CC), periaqueductal gray matter (PAG), red nucleus (RN), and superior colliculus (SC).

### 2.3 Grid dissection of midbrain slices

**Figure 1:**
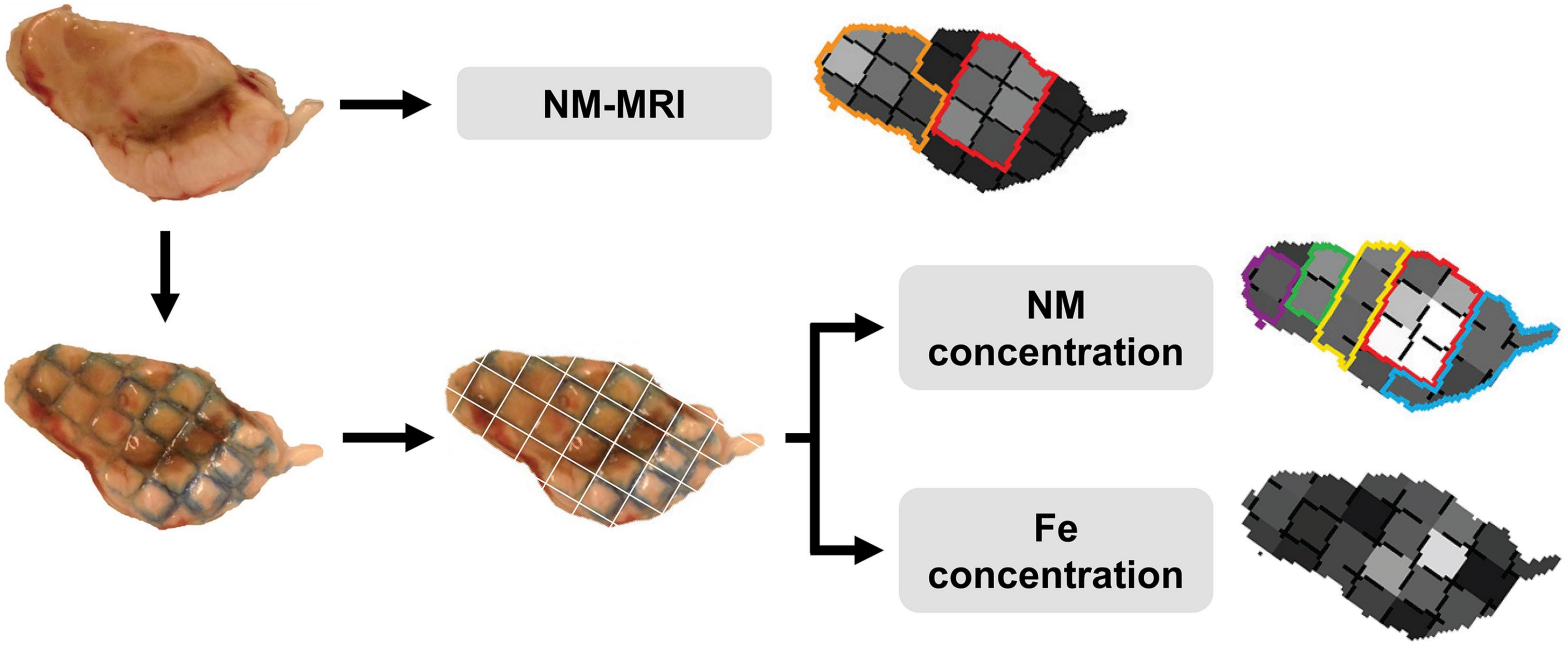
Schematic overview of the experimental steps. Slices of human hemi-midbrain were scanned by using NM-MRI sequences to image NM. After the scanning sessions, the slices were marked with methylene blue using the grid insert as a stamp and dissected along the gridlines to obtain small sections for measurements of NM and Fe concentrations. The three midbrain images on the right (the NM-MRI, the NM and Fe concentration maps) are shown for illustrative purposes only. Gray scale represents signal intensity for NM-MRI and the concentration levels of NM or Fe (see legend of Figure 2).

Within four days post scanning, partially thawed specimens were dissected along gridlines after extensive removal of Fomblin^®^ by dripping tissue slices, followed by gently rolling the surface of the sections on ultraclean filter paper. The slices were dissected in cubes for the determination of NM and Fe concentrations in the tissue (see Figure 1 for experimental steps).

Dissection and manipulation of tissue sections were performed using ceramic blades, and titanium or plastic forceps to avoid Fe contamination. Each specimen was thus divided into 13–36 grid sections; the grid column and row numbers of each dissected section were coded. Each grid section (3.5 mm × 3.5 mm × ~3 mm, depending on the slice thickness), together with any adjacent partial grid section, was weighed, stored separately in plastic tubes, and frozen for the determination of NM and Fe concentrations.

### 2.4 Determination of NM and Fe concentrations in post mortem midbrain slices

Samples derived from each grid section were carefully homogenized with titanium tools. The NM concentration of each grid section was then measured (mostly *n* = 2–4) according to our previously described spectrophotometric method (Zecca et al., 2002), with modifications to improve the removal of interfering tissue components from midbrain regions with higher fiber content and fewer NM-containing neurons compared with the sections of SN proper dissected along anatomical boundaries. Additional tests confirmed that our methods for Fomblin^®^ cleaning were effective and that neither this substance nor the methylene blue dye was likely to influence the spectrophotometric measurements of NM concentration (Cassidy et al., 2019). Data from ~1% of the grid sections (2–3 out of 221) could not be used due to technical problems with dissection, handling, or measurements.

The brain homogenates obtained from each grid section were also used for Fe determination (mostly *n* = 2). To this end, each sample, exactly weighted, was treated according to a previously published method (Ferrari et al., 2017), and analyzed for Fe using inductively coupled plasma-optical emission spectroscopy (ICP-OES, PerkinElmer Optima 3,300 DV; Waltham, MA, USA). The instrumental detection and quantification limits, calculated from linear regression parameters, were 5 and 15 μg/L, respectively. The method detection and quantification limits were 2.5 and 7.5 ng/mg of tissue, respectively. Trueness was estimated using a control sample spiked at two Fe levels (25 and 50 ng/mg), obtaining a quantitative mean recovery of 107% (relative standard deviation 3%; *n* = 3). The repeatability and reproducibility of the results, evaluated on two control samples and calculated as relative standard deviation, were 6% (*n* = 6) and 11%–17% (*n* = 8), respectively.

Total inorganic Fe is stable in the post mortem brain tissue. In addition, the NM was reported to be stable during storage at −15 °C for at least 10 months (Shima et al., 1997). Therefore, the preservation conditions, as reported in Supplementary Table S1, did not affect the NM or Fe concentrations measured in each grid section.

### 2.5 MRI measurement of NM signal in post mortem tissue

Examples of MRI scan quality are reported in Supplementary Figure S2, which shows unprocessed NM-MRI images of two representative hemi-midbrain specimens: one from a subject with AD and another from a subject with PD. NM-MRI signal was measured in the corresponding grid sections using a custom Matlab script, similar to that described in our prior work (Cassidy et al., 2019). Processing of NM-MRI included the automated removal of voxels showing edge artifacts or signal dropout, averaging over slices to create a 2D image, and registration with a grid whose dimensions matched with those of the grid insert. The grid registration was manually adjusted based on the well markers and grid-shaped edge artifacts present in the superior-most slice where the grid inserts rested. The signal in the remaining voxels was averaged within each grid section. A detailed description of these steps follows.

Processing of NM-MRI included the automated removal of low-signal voxels, including both voxels outside of the specimen or those within the specimen showing signal dropout. The threshold for exclusion of low-signal voxels was determined for each specimen based on the distribution of signal across all voxels in the image, which was fitted using a kernel smoothing function. This threshold was set on the smoothed histogram as the minimum lying between two modes: the mode to the left representing voxels outside of the specimen and the mode to the right representing voxels within the specimen. Following this step, a 2D flattened image was generated by averaging over slices.

To eliminate edge artifacts, the first step involved defining the boundaries between the specimen and the surrounding space outside the specimen, as well as between the specimen and areas of signal dropout. To accomplish this, boundary voxels in the flattened image of the specimen that were directly adjacent to low signal voxels (defined above) were labeled using the bwperim function in Matlab. These boundary voxels were dilated by two voxels and subsequently removed indiscriminately from the specimen. To selectively eliminate edge artifacts extending beyond this two-voxel border, a broader border, five voxels thick relative to the original border, was generated and any voxels in this broader border that exhibited contrast-to-noise ratio (CNR) values exceeding 40% were eliminated (note this broader border was not applied to grid sections deemed to contain SN). Outlier voxels exhibiting extreme signal values (Cook’s distance > 4/n in a constant-only linear regression model) relative to other voxels within the same 2D grid section were also removed.

The resulting 2D image, cleaned of edge artifacts, signal dropout, and other outlier voxels, was carried forward to the final processing step, in which CNR was calculated. To normalize signal intensity across specimens, CNR for each voxel ( $\mathrm{CN}R_{v}$ ) in the 2D image was calculated as the relative change in NM-MRI signal intensity ( I ) with respect to a reference region ( RR ) consisting of white matter tracts known to have minimal NM content, the CC subregion, as:


$$\mathrm{CN}R_{v}=(I_{v}-\text{mode}(I_{\mathrm{RR}}))/\text{mode}(I_{\mathrm{RR}})$$

The RR was defined by a region of interest that was manually-traced within the CC on the flattened image of each specimen. The CNR value for each grid section was then calculated as the mean of CNR values of all voxels within the grid section. For grid sections identified through visual inspection as containing SN, the proportion of SN-containing voxels within the grid section was determined by calculating the fraction of grid section voxels with CNR > 10%.

### 2.6 Statistical analysis of post mortem data

A generalized linear mixed-effects model including data across all grid sections ( g ) and specimens ( s ) was used to predict MRI measures in each grid section, that is the mean CNR in a given grid section ( ${\bar{\mathrm{CNR}}}_{\mathrm{gs}}$ ), based on tissue concentration of NM and also Fe in the same grid section. Generalized linear mixed-effects analyses used an isotropic covariance matrix and were fitted *via* maximum pseudo-likelihood estimation, as implemented *via* the Matlab function fitglme. All models included random intercepts but not random slopes ( β) , as:


$${\bar{\mathrm{CNR}}}_{\mathrm{gs}}=\beta_{0}+\beta_{1}\cdot {\left[\bar{\mathrm{NM}}\right]}_{\mathrm{gs}}+\beta_{2}\cdot {\left[\bar{\mathrm{Fe}}\right]}_{\mathrm{gs}}+\beta_{3}\cdot \text{diagnosi}s_{s}+b_{0s}+\varepsilon_{\mathrm{gs}}$$

The model included the mean concentration of NM per grid section ( ${\left[\bar{\mathrm{NM}}\right]}_{\mathrm{gs}}$ ), the mean concentration of Fe per grid section ( ${\left[\bar{\mathrm{Fe}}\right]}_{\mathrm{gs}}$ ), and diagnostic group ( $\text{diagnosi}s_{s}$ ; AD = 0, PD = 1) as fixed-effects predictors, while $\beta_{0}$ is the intercept, $b_{0s}$ are the subject-specific intercepts, and $\varepsilon_{\mathrm{gs}}$ is the error for each grid section. For this analysis a 95% confidence interval was applied for estimations, and the level of statistical significance was set at *p* = 0.050, two-sided.

**Figure 2:**
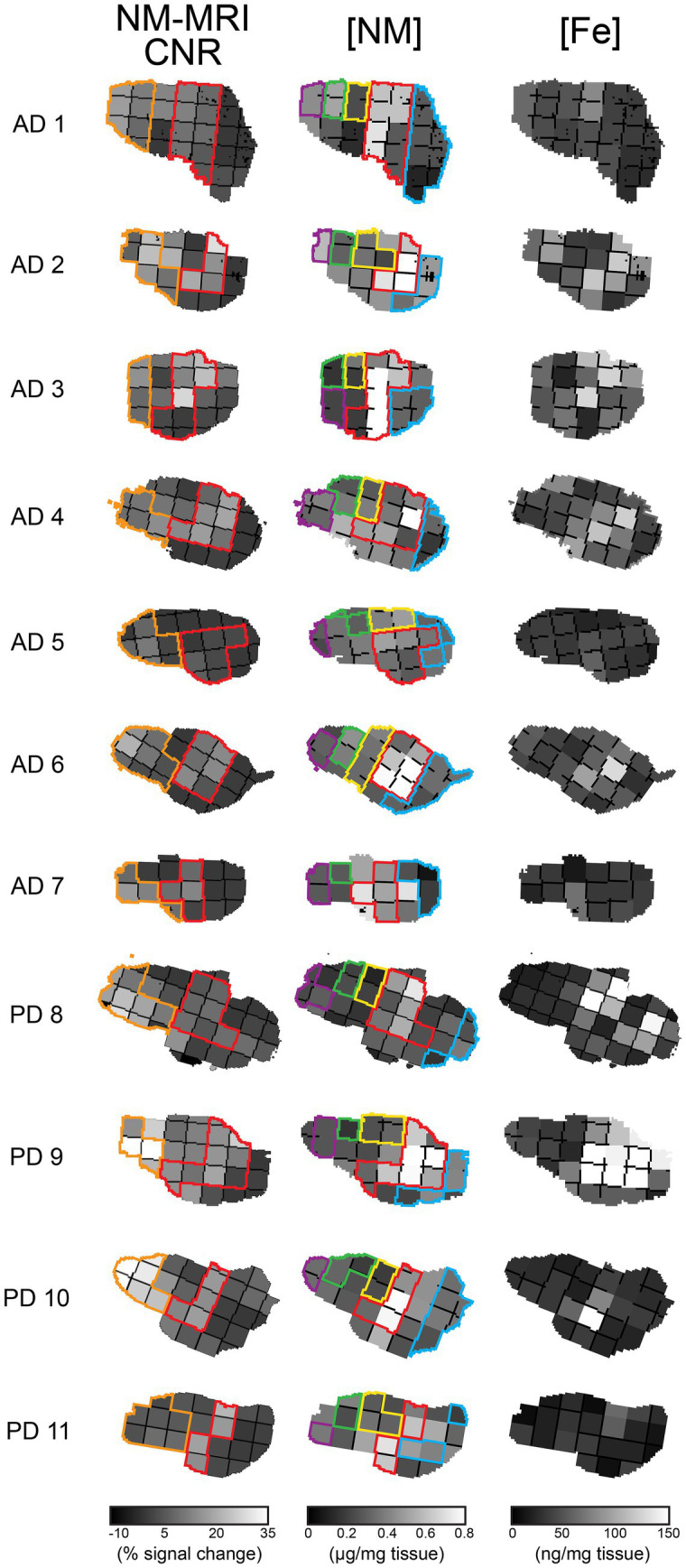
Anatomical topography of NM-MRI and neurochemical measurements for all specimens. Each row represents a different human hemi-midbrain specimen from eleven subjects (seven with AD and four with PD), and each column contains the NM-MRI signal or neurochemical measures (NM and Fe concentrations), respectively. Specimens are divided into grid sections and oriented such that the medial aspect of the hemi-midbrain is at the top and the anterior aspect at the right. Color codes for grid sections: in the first column the posterior midbrain (orange; these grid sections were excluded from analyses relating the NM-MRI signal to NM or Fe concentrations), SN (red); in the second column CC (blue), PAG (green), RN (yellow), SN (red), and SC (purple). CC, crus cerebri; PAG, periaqueductal gray matter; RN, red nucleus; SC, superior colliculus; SN, substantia nigra.

Because our previous findings (Cassidy et al., 2019) indicated that NM-MRI signal was not related to NM tissue concentration in the posterior midbrain (see Figure 2 for definition of this region per specimen), this analysis was conducted separately on grid sections derived from the posterior midbrain and also on those from the remaining (central-anterior) midbrain, the latter being the region of primary interest containing the SN.

A hierarchical linear model within the Bayesian framework was used to test the effect of diagnosis on the three-study metrics (NM-MRI signal, NM concentration, Fe concentration). This model was estimated with Hamiltonian Monte Carlo implemented in Stan v2.21 (Carpenter et al., 2017), and interfaced with BRMS v2.17.0 (Bürkner, 2017) running on R version 4.1.2. Four separate chains with randomized start values each took 3,000 samples from the posterior. The first 1,000 samples from each chain were discarded, so that 8,000 post-warmup samples from the posterior were retained. All chains showed good mixing. R-hat values for all parameters were below 1.01, indicating acceptable convergence between chains. No divergent transitions in any chain were observed. Results on NM and Fe concentrations and on NM-MRI signal across different midbrain subregions of AD and PD subjects, estimated using a hierarchical linear model within the Bayesian framework, are reported in Table 1. This type of analysis allows the relationship between our data and the function of these brain regions, and their impairment in PD compared to AD, thereby revealing cellular changes associated with variations of Fe and NM concentrations. For this analysis, a 95% credible interval was applied for estimations.

**Table 1:** Concentrations of NM and Fe and NM-MRI signal in midbrain subregions of PD and AD subjects.

Region	*n*	NM-MRI signal		Group difference (95% CI)	NM concentration		Group difference (95% CI)	Fe concentration		Group difference (95% CI)
		AD	PD		AD	PD		AD	PD	
SN	60	0.11 ± 0.05	0.15 ± 0.05	0.03 (−0.04 to 0.11)	0.57 ± 0.15	0.61 ± 0.12	0.04 (−0.08 to 0.15)	69 ± 25	103 ± 50	37.3* (11.6 to 64.1)
RN	22	0.05 ± 0.08	0.10 ± 0.07	0.05 (−0.03 to 0.14)	0.33 ± 0.07	0.24 ± 0.07	−0.08 (−0.25 to 0.07)	38 ± 10	38 ± 17	−0.49 (−36.7 to 32.0)
CC	35	0.00 ± 0.03	0.04 ± 0.02	0.04 (−0.04 to 0.12)	0.29 ± 0.09	0.33 ± 0.05	0.03 (−0.11 to 0.17)	42 ± 11	42 ± 25	0.41 (−28.1 to 28.7)
SC	17	0.09 ± 0.09	0.22 ± 0.12	0.12* (0.03 to 0.20)	0.34 ± 0.11	0.24 ± 0.07	−0.09 (−0.27 to 0.08)	54 ± 21	25 ± 7	−26.7 (−62.3 to 9.4)
PAG	18	0.11 ± 0.05	0.32 ± 0.09	0.21* (0.12 to 0.29)	0.35 ± 0.13	0.31 ± 0.06	−0.04 (−0.22 to 0.15)	46 ± 13	33 ± 9	−11.4 (−49.4 to 23.9)

The NM-MRI signal is expressed as CNR, the concentration of NM in μg/mg of tissue and the concentration of Fe in ng/mg of tissue. Reported values are mean ± standard deviation. Group differences and credible interval (CI) are estimated by a linear model within the Bayesian framework. (*) Statistically significant. CC, crus cerebri; PAG, periaqueductal gray matter; RN, red nucleus; SC, superior colliculus; SN, substantia nigra.

## 3 Results

### 3.1 Topography of NM and Fe content in human midbrain

Subregions of human hemi-midbrain specimens (Supplementary Figure S1) were scanned using NM-MRI and then dissected into grid sections in which NM and Fe concentrations were determined (see Figure 1 for experimental steps). Typical unprocessed NM-MRI images of hemi-midbrain slices are shown in Supplementary Figure S2. In these images, the SN is clear in the AD subject, confirming the presence of dopamine pigmented neurons, and has a lower signal in PD, due to the loss of NM-containing neurons.

The anatomical topography of all these measures is shown in Figure 2. The first column shows outlines of grid sections labelled as the SN and the posterior midbrain, the latter of which was excluded from analyses relating NM-MRI signal to NM and Fe concentrations. The second column shows outlines of grid sections labelling the SN and neighboring regions analyzed in this study (see legend for color codes): CC, PAG, RN, and SC.

### 3.2 Relationship between tissue NM and Fe content and NM-MRI measures

**Figure 3:**
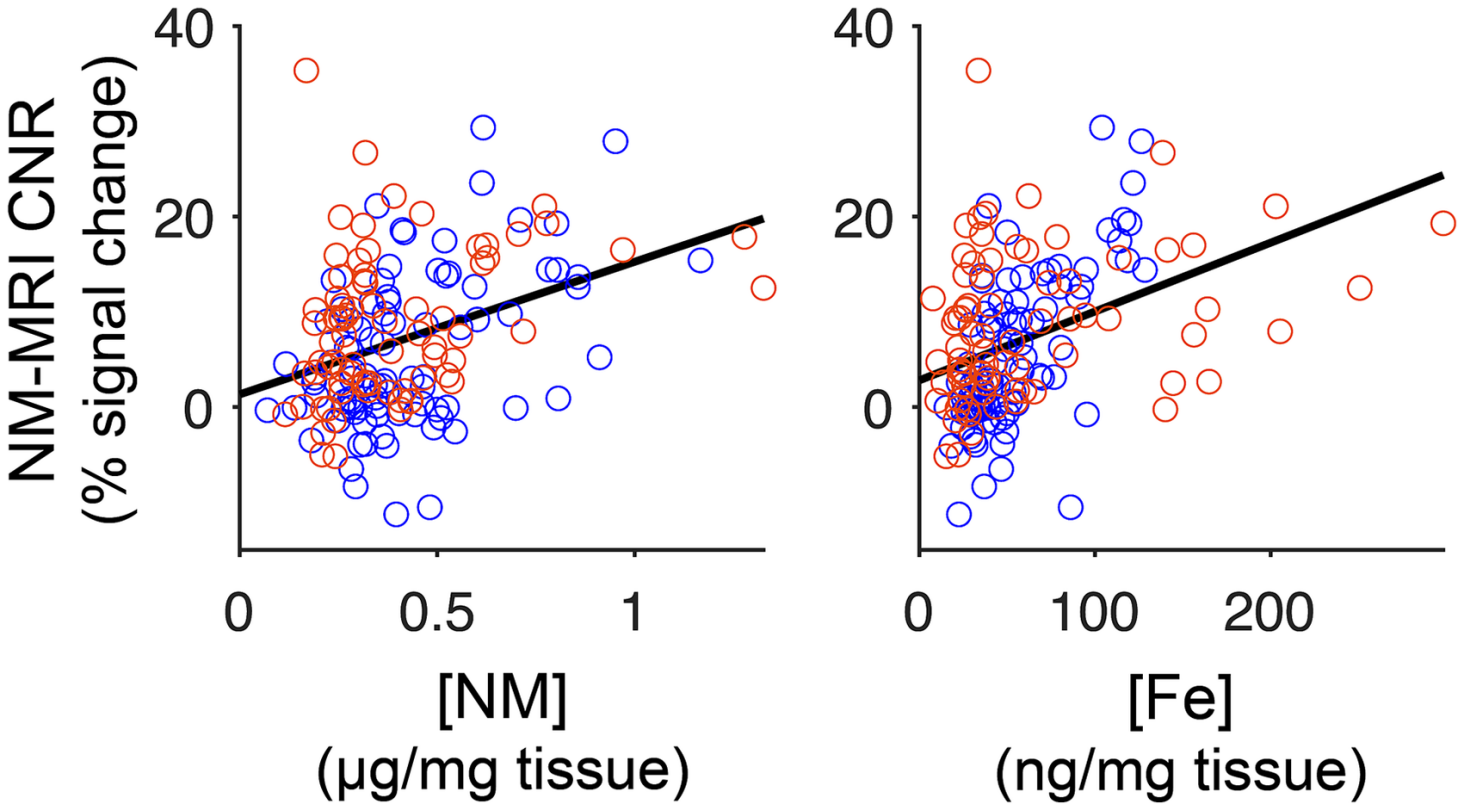
Correlation between NM or Fe concentrations and the NM-MRI signal. Scatterplots showing the positive correlation between NM concentration and the NM-MRI signal (left; *β*_1_ = 7.37, with 95% confidence interval = 1.26 to 13.50) and between Fe concentration and the NM-MRI signal (right; *β*_2_ = 0.050, with 95% confidence interval = 0.019 to 0.080). The *R*^2^ for the model including both Fe and NM concentrations is 0.30. Measures derived from AD specimens are shown in blue (*n* = 87) and those from PD specimens in red (*n* = 76).

Our primary objective was to determine how variability in the NM-MRI signal depends on the tissue content of NM and Fe. We examined the independent contributions of NM and Fe tissue concentrations to the NM-MRI signal in midbrain grid sections. The analysis model included NM concentration per grid section, Fe concentration per grid section, and diagnostic group as fixed-effects predictors. Our main finding was that both NM concentration (*β*_1_ = 7.37, with 95% confidence interval = 1.26 to 13.50, *t*_159_ = 2.38, *p* = 0.018) and Fe concentration (*β*_2_ = 0.050, with 95% confidence interval = 0.019 to 0.080, *t*_159_ = 3.17, *p* = 0.0018) provided unique significant contributions to the NM-MRI signal (mixed-effects model controlling for diagnosis, 163 grid sections, 11 specimens; Figure 3). This analysis excluded grid sections in the posterior midbrain (Figure 2), as we have previously observed the NM-MRI signal to be unrelated to the tissue content of NM in that region (Cassidy et al., 2019). Indeed, in the posterior midbrain, neither NM (*p* = 0.82) nor Fe (*p* = 0.083) concentrations were significantly related to the NM-MRI signal (mixed-effects model controlling for diagnosis, 56 grid sections).

### 3.3 Concentrations of NM, Fe, and NM-MRI signal in midbrain subregions of AD and PD subjects

Our second objective was to compare the imaging and neurochemical measures across specific brain subregions between PD and AD samples. Consistent with the loss of NM-containing SN neurons in PD (Pakkenberg et al., 1991; Kastner et al., 1992), we expected the SN-containing grid sections of PD samples to have lower NM content than those of AD samples: however, with this small sample size, we did not find a significant difference between the two groups in NM content (Table 1). Exploratory analyses of SN-neighboring regions examining NM content, Fe content, and NM-MRI signal found statistically significant differences, estimated using a hierarchical linear model within a Bayesian framework, in NM-MRI signal in the PAG (estimated group difference = 0.21, with 95% credible interval = 0.12 to 0.29) and the adjacent SC (estimated group difference = 0.12, with 95% credible interval = 0.03 to 0.20), both of which elevated in the PD group compared to the AD group. A significant difference in Fe concentration was found in the SN, with a higher level in the PD group than in the AD group (estimated group difference = 37.3, with 95% credible interval = 11.6 to 64.1).

## 4 Discussion

An important objective of this study was to determine the independent influences of NM and Fe contents on the NM-MRI signal. We found that the tissue content of NM influences the NM-MRI signal independently from the tissue content of Fe, which exerts its own influence. This finding could be interpreted in different ways: it may suggest that there are important properties of NM, unrelated to its Fe content, that influence the NM-MRI signal; alternatively, it could suggest that NM concentration serves as a proxy measure of NM-bound Fe, which could be the most important factor influencing NM-MRI signal. Indeed, it was shown that the intraneuronal NM-Fe complex is paramagnetic, whereas ferritin has a very low paramagnetic effect but is present at higher concentration than NM in glial cells (Zecca et al., 2004). NM contains a stable free radical and is therefore paramagnetic itself, like the NM-Fe complex (Shima et al., 1997).

We prepared a detailed map of NM and Fe concentrations in subregions of the human midbrain from AD and PD subjects, including SN and neighboring regions like CC, PAG, RN, and SC. To date, Fe and NM concentrations have been reported mainly in the SN (Zecca et al., 2004; Zecca et al., 2008a). In NM-containing neurons of the SN, most of the Fe is present in NM-Fe complex (up to ~11 μg Fe/mg of isolated NM pigment), while ferritin-bound Fe is mainly located in glial cells (Zecca et al., 2004; Shima et al., 1997; Zecca et al., 2008b). Despite the correlation observed here between NM-MRI signal and actual Fe concentration, a recent study found NM-MRI signal not to correlate with a common MRI Fe measurement, namely quantitative susceptibility mapping (Chen et al., 2023). We report here that other midbrain gray regions and white matter contain NM and Fe concentrations lower than those of SN.

We previously reported, in a model system, that synthetic NM-Fe complexes showed an increase of R_1_ (and T_1_ shortening) with increasing Fe-bound to NM models, an effect stronger than the influence of Fe alone, supporting the role of the NM-Fe complex in generating the MRI contrast of the SN (Trujillo et al., 2017). In addition, this study demonstrated that the longitudinal and transverse relaxation rates of the melanic component of NM models, which is bound to Fe, were linearly related to the concentration of the melanic component when Fe was bound. Indeed, in this synthetic model, NM did not influence relaxation times in the absence of Fe (Trujillo et al., 2017). We can reconcile our current findings with this past work if we consider that the concentration of NM in the present study may serve to estimate the portion of Fe that is NM-bound, which may have a particularly strong impact on NM-MRI signal, above that accounted for by our measure of total Fe concentration, which includes NM-bound Fe as well as other Fe sources like ferritins and additional molecules (Zecca et al., 2004).

We did not find the expected decrease of NM concentration in the SN of PD patients, resulting from the death of NM-containing dopamine neurons (Pakkenberg et al., 1991; Kastner et al., 1992; Zecca et al., 2002), when compared with SN grid sections of AD tissues, where the loss of NM-containing neurons is mild and variable (Zarow et al., 2003), leading to unchanged NM concentration levels compared to healthy controls (Cai et al., 2023). This is likely a false negative finding due to a limitation in our study, that is the low number of subjects, leading us to be underpowered to observe a significant difference in the NM content in the SN between PD and AD subjects. Another limitation of our study is the absence of detailed information about the donors of the samples (e.g., neuropathological status and medication information). In addition, inconsistencies in the preparation of post mortem specimens might have contributed. However, in PD subjects, compared to AD, we found the expected significant increase of Fe concentration in grid sections containing the SN, consistent with previous findings (Dexter et al., 1989; Riederer et al., 1989; Jellinger et al., 1992; Brooks et al., 2020). The absence of changes in Fe concentrations in the RN is consistent with previous neurochemical investigations in post mortem RN of PD subjects (Riederer et al., 1989). These results, obtained using accurate spectroscopy methods on dissected RN, are in contrast with the increased Fe deposition reported in the RN of PD subjects compared with controls using MRI methods, although MRI results remain widely debated (Wang et al., 2016). Moreover, various Fe-containing compounds, such as ferritins, hemosiderin, NM, and others with different magnetic characteristics, are present in PD brains in amounts that differ from those in controls, and this may explain inconsistencies among different MRI studies (Reimão et al., 2016; Wang et al., 2016).

An increase of NM-MRI signal was observed in the PAG and adjacent SC of PD subjects compared to those with AD, without a corresponding increase of NM or Fe concentrations, perhaps suggesting an increase in the fraction of water protons relative to macromolecular protons (Trujillo et al., 2017, 2024). Combining NM-MRI signal measures from the SN and PAG may be useful for optimizing NM-MRI as a diagnostic biomarker for PD, since the PAG is affected in this disease (Kumakura et al., 2010). In PD, Lewy bodies and neurites are found with alpha-synuclein immunoreactive neuronal and oligodendroglial aggregates in the PAG and other brainstem structures, indicating involvement of the PAG in PD pathology (Seidel et al., 2015). In PD subjects, alterations in sensory function, particularly in pain perception, might result from PAG involvement (Conte et al., 2013).

The SC has been suggested to be damaged early in PD, before the SN, with invasion of alpha-synuclein aggregates (Braak et al., 2003). In subjects with PD, the interneurons of the SC, which are targeted by projections from the SN, are abnormally inhibited, and such inhibition may contribute to PD symptoms like visual deficits, including perceptual and attentional impairments, and hallucinations (Pretegiani et al., 2019).

Several studies have shown a progressive decrease in NM-MRI signal and SN volume in PD patients compared with healthy controls; therefore, this method could become a valuable biomarker for confirming the diagnosis of PD and for monitoring disease-modifying treatments (Biondetti et al., 2020; Reimão et al., 2020; Gaurav et al., 2021; Trujillo et al., 2024). If replicated, the NM-MRI signal increase we observed in the PAG and SC of patients with PD could be further investigated for its potential as a biomarker for the early diagnosis of PD, including in subjects with prodromal symptoms or a family history of the disease.

In conclusion, this study provides a detailed map of NM and Fe concentrations in the midbrain subregions of PD and AD patients, demonstrating that both NM and Fe provide unique significant contributions to the NM-MRI signal. We also demonstrated the utility of NM-MRI in detecting NM and Fe to enhance the robustness of live-subject studies and strengthen clinical correlations. The possibility of monitoring NM and Fe as biomarkers remains, to date, the most promising tool for the diagnosis and monitoring of treatment in PD and other movement disorders.

## Data Availability

The raw data supporting the conclusions of this article will be made available by the authors, without undue reservation.
