# Supplementary material for: Neuromelanin, iron and MRI measurements in midbrain tissues of Parkinson’s and Alzheimer’s subjects
Source: Front Aging Neurosci. 2026 Feb 4;18:1672578. doi: 10.3389/fnagi.2026.1672578 (PMC12913387; doi:10.3389/fnagi.2026.1672578)
Supplement: Supplementary file 1 [file Data_Sheet_1.docx]

Supplementary Material

**Supplementary Figures**


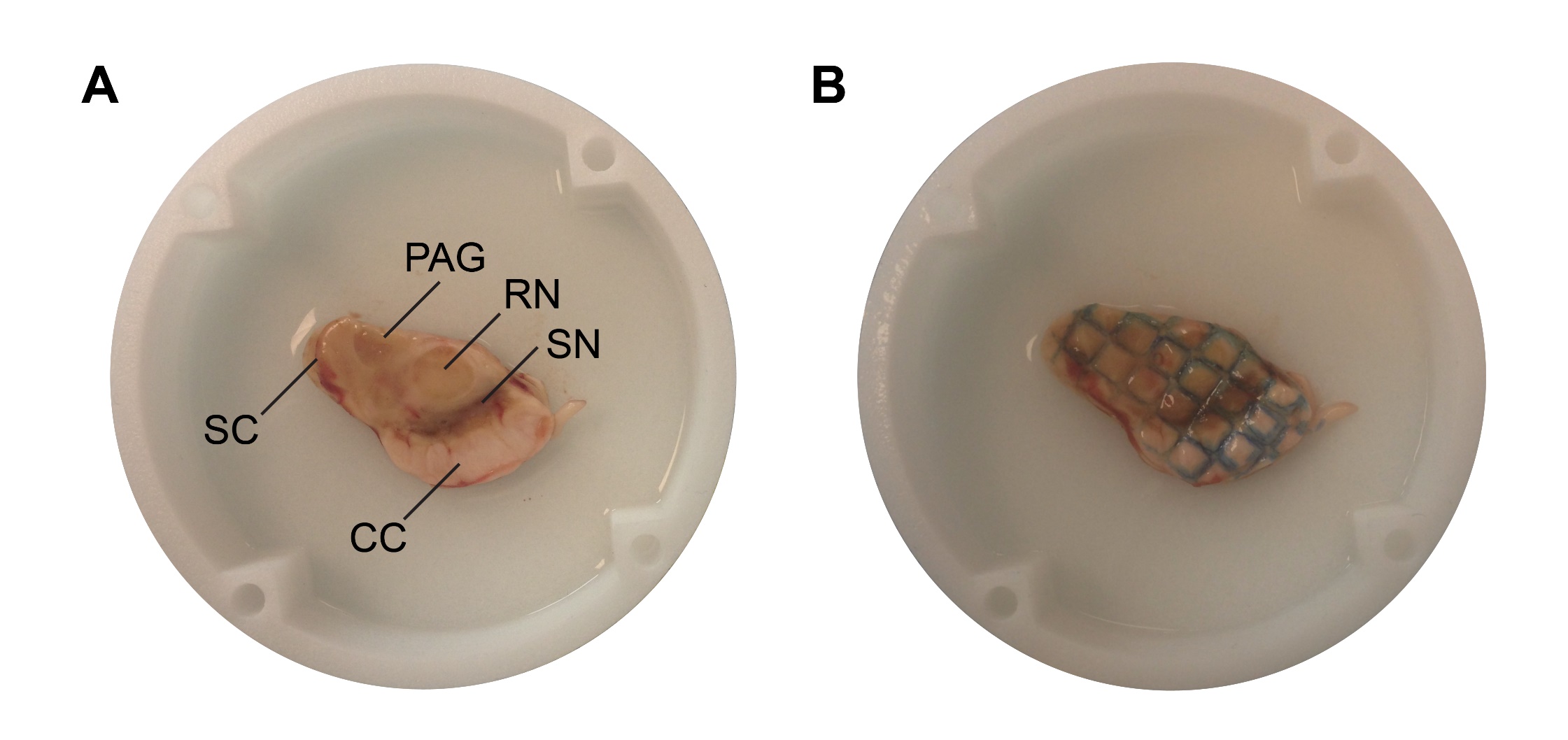


**Supplementary Figure 1.** Photographs of representative hemi-midbrain specimen in the MRI-compatible dish. Clearly visible structures are labeled in panel A, while panel B shows the same specimen (subject AD 6 in Fig. 1 of the main text) after being stamped with the grid-insert to demarcate gridlines for dissection. CC, crus cerebri; PAG, periaqueductal gray matter; RN, red nucleus; SC, superior colliculus; SN, substantia nigra.


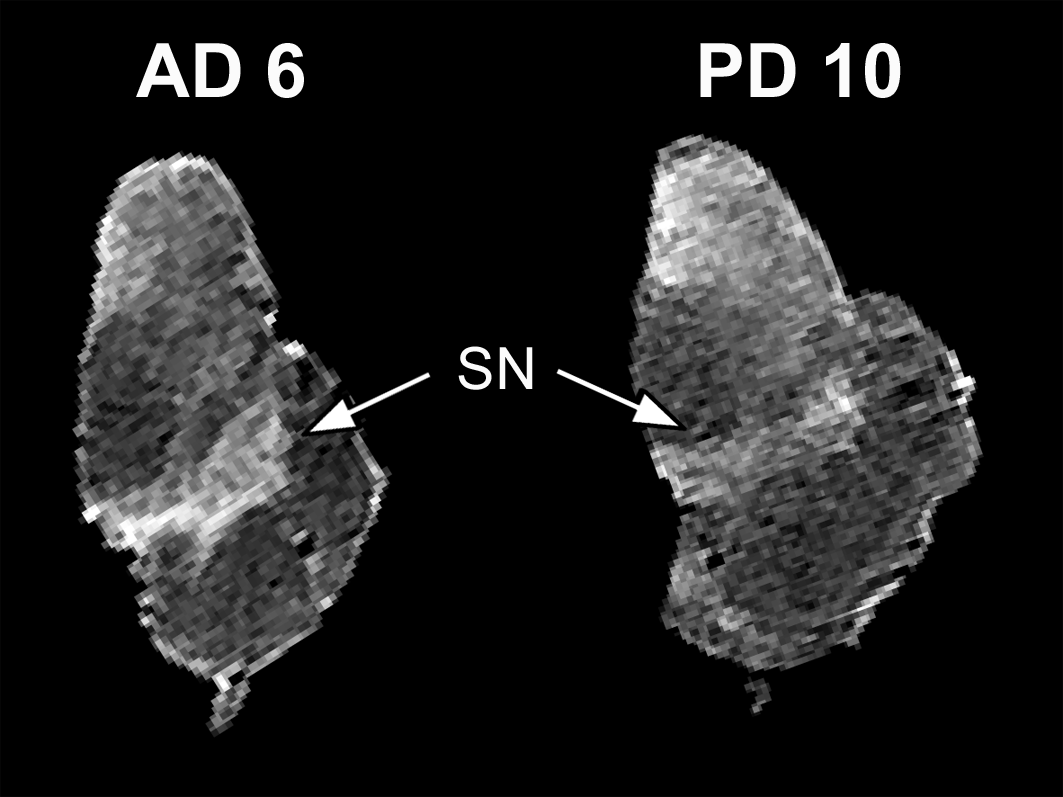


**Supplementary Figure 2.** Examples of unprocessed NM-MRI images. This figure shows raw NM-MRI scans of two representative hemi-midbrain specimens from one subject with AD and another with PD (subjects AD 6 and PD 10 in Fig. 1 of the main text).

**Supplementary Table**

**Supplementary Table 1. Demographic and clinical information of subjects.**

| Sample ID | Age | Sex | Clinical diagnosis | Neuropathological diagnosis | Cold post mortem interval (hours) | Frozen post mortem interval (hours) | Disease duration (years) |
| --- | --- | --- | --- | --- | --- | --- | --- |
| 1 | 81 | F | AD | AD | 6.5 | 19.3 | - |
| 2 | 76 | F | AD | AD | 5.5 | 18.2 | - |
| 3 | 44 | M | AD | AD | 2.8 | 20.8 | - |
| 4 | 72 | M | AD | AD | - | 9.0 | - |
| 5 | >88 | - | AD (possible) | AD | 3.1 | 25.7 | - |
| 6 | >88 | F | Dementia | AD | 0.8 | 16.5 | - |
| 7 | 84 | F | AD (possible) | AD | 1.8 | 23.7 | - |
| 8 | 83 | M | PD with dementia | Diffuse Lewy body disease, limbic or transitional type | 2.8 | 20.8 | 6 |
| 9 | 67 | M | PD with dementia | Diffuse Lewy body disease, limbic or transitional type | 3.0 | 12.5 | 14 |
| 10 | 79 | M | PD with dementia | Diffuse Lewy body disease | - | 9.0 | - |
| 11 | 83 | M | PD with dementia | Diffuse Lewy body disease, cortical type | 0.0 | 36.75 | 18 |

Note that the younger male subject (aged 44 years) was retained despite his substantial age difference, as his data were within the same range as those of the other subjects, and the exclusion of this subject had little impact on the results. AD, Alzheimer’s disease; F, female; M, male; PD, Parkinson’s disease.
